# The Efficacy of Buprenorphine in Major Depression, Treatment-Resistant Depression and Suicidal Behavior: A Systematic Review

**DOI:** 10.3390/ijms19082410

**Published:** 2018-08-15

**Authors:** Gianluca Serafini, Giulia Adavastro, Giovanna Canepa, Domenico De Berardis, Alessandro Valchera, Maurizio Pompili, Henry Nasrallah, Mario Amore

**Affiliations:** 1 Department of Neuroscience, Rehabilitation, Ophthalmology, Genetics, Maternal and Child Health, Section of Psychiatry, University of Genoa, 16132 Genoa, Italy; siriogiulia@libero.it (G.A.); giovanna.canepa@ordinepsicologiliguria.it (G.C.); mario.amore@unige.it (M.A.); 2 IRCCS Ospedale Policlinico San Martino, 16132 Genoa, Italy; 3 Villa San Giuseppe Hospital, Hermanas Hospitalarias, Ascoli Piceno, Italy, Polyedra Research Group, 64100 Teramo, Italy; dodebera@aliceposta.it; 4 NHS, Department of Mental Health, Psychiatric Service of Diagnosis and Treatment, Hospital “G. Mazzini”, Asl 4, 64100 Teramo, Italy; a.valchera@ospedaliere.it; 5 Department of Neurosciences, Mental Health and Sensory Organs, Suicide Prevention Center, Sant’Andrea Hospital, Sapienza University of Rome, 00189 Rome, Italy; maurizio.pompili@uniroma1.it; 6 Department of Neurology & Psychiatry, Saint Louis University School of Medicine, St. Louis, MO 63104, USA; hnasral@slu.edu

**Keywords:** endocannabinoid system, buprenorphine, treatment-resistant depression, major depression, suicidal behavior

## Abstract

Although several pharmacological options to treat depression are currently available, approximately one third of patients who receive antidepressant medications do not respond adequately or achieve a complete remission. Thus, novel strategies are needed to successfully address those who did not respond, or partially respond, to available antidepressant pharmacotherapy. Research findings revealed that the opioid system is significantly involved in the regulation of mood and incentives salience and may be an appropriate target for novel therapeutic agents. The present study aimed to systematically review the current literature about the use of buprenorphine (BUP) for major depression, treatment-resistant depression (TRD), non-suicidal self-injury (NSSI) behavior, and suicidal behavior. We investigated Pubmed and Scopus databases using the following keywords: “buprenorphine AND depression”, “buprenorphine AND treatment resistant depression”, “buprenorphine AND suicid*”, “buprenorphine AND refractory depression”. Several evidence demonstrate that, at low doses, BUP is an efficacious, well-tolerated, and safe option in reducing depressive symptoms, serious suicidal ideation, and NSSI, even in patients with TRD. However, more studies are needed to evaluate the long-term effects, and relative efficacy of specific combinations (e.g., BUP + samidorphan (BUP/SAM), BUP + naloxone (BUP/NAL), BUP + naltrexone) over BUP monotherapy or adjunctive BUP treatment with standard antidepressants, as well as to obtain more uniform guidance about the optimal BUP dosing interval.

## 1. Introduction

### 1.1. The Impact of Major Depression and Treatment Resistant Depression Worldwide

According to WHO estimation, more than 320 million people are affected by major depression worldwide, with the prevalence of this disabling condition that is increased by 18.4% from 2005 to 2015 [1]. Depression may be considered the second leading cause of disability (7.5% of all Years Lived with Disability—YLD) [2]. Although the burden and disability related to this condition, the STAR*D study clearly showed that about 50% of patients with major depressive disorder (MDD) will experience a response with the first treatment [3] but only 30% achieve a complete remission and the remaining percentage will need to undergo several additional treatment trials in order to improve response [4]. These remission rates are lower in treatment-resistant depression (TRD) [5] which is associated with a higher risk of recurrence, substance abuse, and suicidal behavior [6,7]. Furthermore, MDD frequently appears to exert neuro-progressive clinical characteristics, with recurring episodes of increasing severity, reduced therapeutic response [8], and persistence of residual symptoms which commonly predict a poorer outcome [9]. In this context, it is crucial to obtain a complete remission or—at least—manage and appropriately treat depressive residual symptoms in order to reduce the risk of relapses, associated psychosocial impairment, and suicidal attempts [6,10].

Importantly, while suicidal behavior occurs in the presence of any psychiatric condition, consistent studies repeatedly showed that suicide is most common in subjects with major mood disorders (MDD and bipolar disorder) [11]. In 2015, suicide across all ages entered the top 20 leading causes of death—accounting as the most relevant cause of death for 1.5% of all deaths worldwide—and the second one among 15–29 years old after accidents [2]. These numbers are underestimated in case we take into account those who made nonfatal suicide attempts and those who experienced suicidal ideation or engaged in non-suicidal-self-injury-behavior (NSSI) [2].

NSSI was recently added to the Diagnostic and Statistical Manual of Mental Disorders—5th edition (DSM 5), and it is commonly defined as the intentional, direct destruction of body tissue without suicidal intent [12]. It has a prevalence of 4% in the general population and up to 21% in clinical samples [13]; this behavior doubles the expected all-cause mortality rate (risk of death from suicide, accidents, and natural causes) of subjects who engage in it compared with the general population [14]. Endogenous opioid system seems to be involved in the etiology of NSSI behavior too, as suggested by the efficacy of opioid antagonists (i.e., naloxone and naltrexone) in reducing these behaviors. Other effective treatment options for NSSI are needed, though, in particular for non-responders [15]. 

### 1.2. Psychoacvtive Treatments for Major Affective Disorders: A Brief Overview

The use of *Papaver somniferum* derivatives as a cure for various ills goes back to prehistory; opioid cures for melancholia was proposed in the early 1900s, with the use of this compound—although seemingly effective—which has been slowed by its high addictive properties [16]. In the 1950s, the discovery of euphoric properties of isoniazid—a monoamine oxidase inhibitor’s (MAOi) progenitor—and imipramine—from which the tricyclics derive—gradually reduced the importance of opiates [17], and currently selective serotonin reuptake inhibitors (SSRIs) are considered the standard first-line treatment for major depression [18,19]. Unfortunately, as we stated above, SSRIs are effective in only 40–50% of patients [20]; thus, currently, opioids are being re-investigated in order to create new therapeutic options for major depression with a reduced abuse potential, e.g., using the combination with compounds such as naloxone or samidorphan [21,22].

From a neurobiological point of view, opioids play a crucial role in pain processing, stress responses, respiration, gastrointestinal transit, and the endocrine system—in particular, the hypothalamus-pituitary-adrenal gland (HPA) axis—and immune functions [23,24], and their dysregulation exerts an important role in attachment, loss, anhedonia, and MDD itself [16,25]. The endogenous opioid system is composed of three different G-protein coupled receptors (GPCRs)—µ-, δ-, and κ-opioids receptors (MORs, DORs, and KORs, respectively)—that are linked with a family of endogenous opioid peptides known as β-endorphin, enkephalins, and dynorphins [26]. These receptors are widespread in central and peripheral nervous system, with a high density in limbic areas that explains, at least partially, their role in reward processing and mood control, and supports their use to treat emotional dysfunction [27]. These pharmacological options have been hypothesized to modulate BDNF activity and enhance neurogenesis in the hippocampus as well [28].

Preclinical research with constitutive knockout (KO) mice showed that MORs, DORs and KORs have distinct role over mood-related processes [29]. In particular MOR—in which BUP acts as a partial agonist—is a key molecular player in the reward processing circuit contributing to recreational drug use and addictive behaviors. It has been reported that MOR activation in the dorsal raphe nucleus (DRN) and ventral tegmental area (VTA) by local GABAergic interneurons disinhibits both 5-HT and DA neurons, but inhibits noradrenergic neurons [26,30,31]. The MOR-mediated mechanism of mood control is more complex than described above; in fact, some studies showed even a paradoxical depressive-like potential of MOR according to the evidence that two groups of MOR KO mice appeared to have decreased anxiety- and depressive-like behaviors [32]. DOR—that is antagonized by BUP—together with encephalin, seems to have a mood-enhancing activity, but it is not clear how it regulates the reward process [33]. KOR—antagonized by BUP, too—exhibits, in contrast to MOR activity, a major anti-reward role being able to reduce reward tonically [34]. Interestingly, this type of activity is potentiated by different stressors and may play a role in various stress-induced psychopathological conditions [24]. The dynorphin/KOR system, through the action on DA neurons in the nucleus accumbens (NAc), may be linked to depressive-like behaviors [24].

### 1.3. The Potential of Buprenorphine

Pharmacodynamic notions: BUP is a MOR partial agonist and a KOR/DOR antagonist; it binds with a high affinity to MOR and KOR, and it binds with a lower affinity to DOR [35].

By a pharmacokinetic point of view, BUP, due to the first-pass effect, has a low oral bioavailability and, therefore, a sublingual administration (bioavailability of almost 51%) seems more useful [36]. The peak serum concentration after multiple doses of BUP is reached in 1 to 2 h, approximately, while the time needed to reach the maximum concentration (Tmax) after a single dose, is about 40 min to 3.5 h [37].

Concerning metabolism and elimination, BUP undergoes hepatic metabolism—primarily by CYP450-3A4 and CYP 2C8—and, after *N*-dealkylation, it is transformed in nor-BUP [38]. These two compounds are then glucuronized [39], and later excreted by the renal and biliary route. About 70% of the drug is fecally excreted; however, it is re-absorbed as free BUP, and nor-BUP [39].

As mentioned previously, depression is a high-burden disease, in terms of YLD, with a high prevalence of recurrence and clinicians have to commonly face a relevant issue in its pharmacological treatment with typical drugs that impact on monoamine system. Given this background, it is important to find other compounds that act on different systems; BUP is a synthetic opiate that has shown, in both animal models and human studies, interesting antidepressant-like effects, and a reduction of self-injurious behavior and suicidal behavior [40].

Thus, the present report is mainly aimed to perform a systematic review of the current literature about BUP in major depression, TRD, NSSI, and suicidal behavior. 

## 2. Results

### 2.1. Study Sample

**Figure 1:**
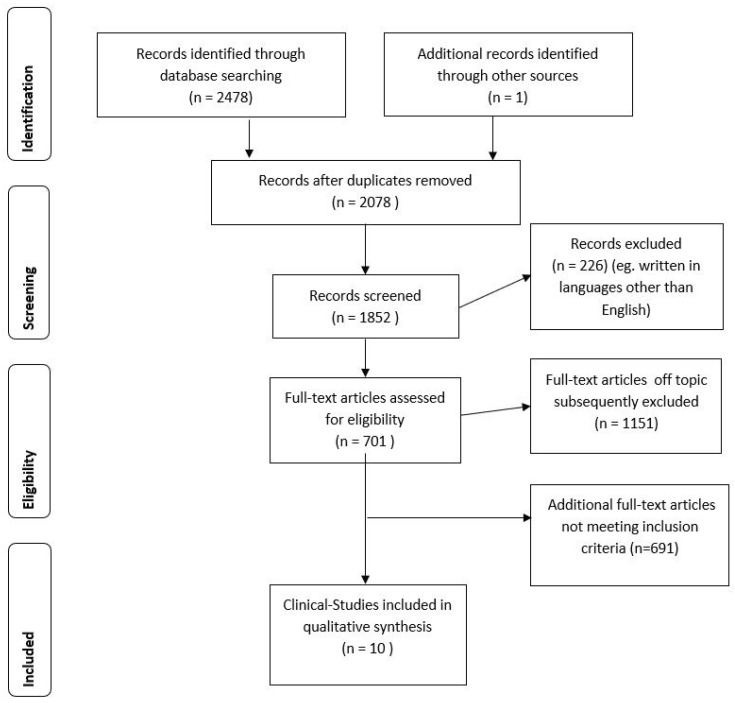
Stages of the screening process about BUP and depression.

The searches in Pubmed and Scopus databases revealed, after the removal of duplicates, a total of 2478 potentially relevant articles about BUP and depression (e.g., unipolar and bipolar depression, TRD). Overall, the search generated 478 articles in Pubmed and 2000 in Scopus, respectively. One additional record was identified through other sources. After the removal of duplicates, a total of 2078 potentially relevant articles about BUP and depression (e.g., unipolar and bipolar depression, TRD) remained. Of these, 2068 were excluded because they were without an abstract or had an abstract that did not explicitly mention depression or suicidal ideation, or were on animal studies, or were written in a non-English language. Thus, 10 studies met our inclusion criteria and were included in the present review. Figure 1 summarizes the main results of the search strategy (identification, screening, eligibility, and inclusion process) used for selecting studies.

**Figure 2:**
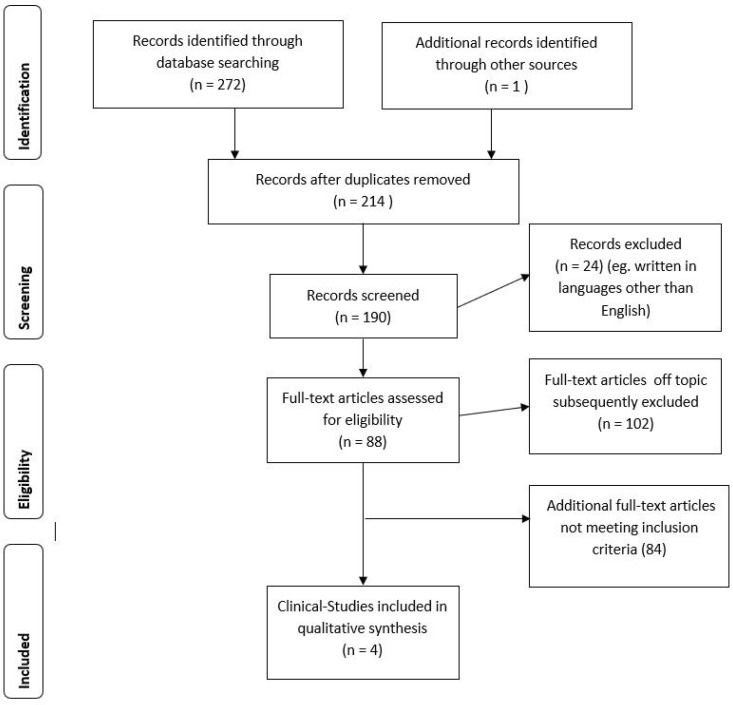
Stages of the screening process about BUP and suicidal behavior.

Concerning BUP and suicidal behavior (e.g., suicidal ideation, suicidal thoughts, suicide attempts or NSSI), the search generated 273 articles (65 articles in Pubmed and 208 in Scopus). One additional record was identified through other sources. After the removal of duplicates, a total of 214 potentially relevant articles remained. Of these, 210 were excluded because they were without an abstract or had an abstract that did not explicitly mention suicidal behavior. Thus, four studies met our inclusion criteria and were included in the present review. Figure 2 summarizes the main findings of the search strategy (identification, screening, eligibility, and inclusion process) used for selecting studies.

### 2.2. Study Types and Sample Characteristics

Overall, six clinical trials [41,42,43,44,45,46]—of which three were randomized, double-blind and placebo controlled studies [41,44,45]—and one clinical case [47], including a total of 216 subjects with TRD treated with BUP in mono-therapy or together with an opioid antagonist (naloxone or samidorphan) were included. Three additional studies investigating the course of depressive symptoms in opiate dependence patients treated with BUP were also considered in addition to the Striebel case (a total of 248 patients) [48,49,50].

Regarding the efficacy of BUP on suicidal ideation, in addition to the Striebel report, two other studies were added—one randomized, double-blind, and placebo-controlled clinical trial [51], and one case report [52]—for a total of 90 patients having heterogeneous diagnoses (i.e., Borderline Personality Disorder, Major Depressive Disorder, Adjustment Disorders, Eating Disorders, Post-Traumatic Stress Disorder, Substance-Induced Depressive Disorder), but all with clinically significant suicidal ideation. Only one clinical case study about NSSI behavior was found [53]: it included 6 patients with self-injury behavior who were treated with BUP.

### 2.3. Study Quality Assessment

According to our quality score system, the mean score of the seven studies regarding BUP and TRD was 4.7; the mean score of the four studies on opiate-dependent patients was 3.5; the mean score of the three studies about BUP and suicide ideation was 3; the mean score of the Norelli et al. study about NSSI was 2, respectively. Specifically, the mean score of the three case-report studies concerning BUP and depression/suicidal behavior included in the present review was 2 while the mean score of the ten controlled studies concerning BUP and depression/suicidal behavior included in the present review was 5.

Overall, most of the included studies (N = 8) were of moderate quality, one was of good quality, and four of low quality. The most relevant findings of the thirteen included studies are reported below. The three case-report studies concerning BUP and depression/suicidal behavior were of low quality while the ten controlled studies concerning BUP and depression/suicidal behavior included in the present review were of moderate quality.

### 2.4. Studies Description

Three case-reports were included in this review: the studies of Striebel and Kalapatapu [47], Ahmadi et al. [52], and Norelli et al. [53]. Striebel and Kalapatapu [47] described the case of a 61-year old women affected by chronic-opiate dependent back pain and depression who was treated with 16 mg/4 mg of BUP/naloxone daily. This drug combination was associated with no craving, managed her pain, and improved her mood, arresting suicidal ideation. Moreover, Ahmadi and colleagues [52] investigated a patient with suicidal ideation and substance-induced depression: the 25-year old man was successfully treated with a 8 mg single dose of sublingual BUP as an adjunctive treatment to olanzapine and valproate 20 and 400 mg daily doses, respectively. Furthermore, Norelli et al. [53] described a case series including six psychiatric inpatients with severe, treatment refractory NSSI on which BUP has been administered—in different doses—in combination with other therapies (e.g., antipsychotic medications including clozapine, antidepressants, mood stabilizers including lithium, valproate, and carbamazepine, benzodiazepines, α-adrenergic and β-blockers, and the opiate antagonist naltrexone). Overall, six patients showed an improvement in NSSI behavior. These three case reports demonstrate a relevant efficacy of BUP treatment on suicidal ideation, depression symptoms, and NSSI.

Then, the three observational-prospective studies conducted by Nyhuis and colleagues [46], Kosten et al. [48], and Gerra et al. [50] were assessed. Nyhuis and colleagues [46] observed in six nonpsychotic TRD patients an improvement of depressive symptoms after seven days of BUP treatment, as a monotherapy, at a medium dose of 1.2 mg/day. Kosten et al. [48] and Gerra et al. [50] assessed depressive symptoms in substance dependent patients (40 and 60 subjects, respectively). Gerra and colleagues [50] observed a greater reduction of irritability, depression, tiredness, and psychosomatic symptoms in the BUP + naltrexone group *vs.* naltrexone alone group after 12 weeks of treatment. Kosten et al. reported antidepressant effects during one month of treatment in opioid dependent patients switched to BUP from methadone or treated directly with BUP (mean sublingual daily BUP dose of 3.2 mg).

In this review, we also evaluated two non-controlled, open label clinical trials (Bodkin et al. [42] and Karp et al. [43]). Bodkin [42] treated 10 TRD subjects with BUP, according to tolerance and clinical benefits, with a maximum daily dosage of 1.8 mg. After four to six weeks, seven patients showed clinically striking improvements in both subjective and objective measures of depression. Similarly, Karp et al. [43] found an improvement of depressive symptoms in 15 TRD patients after BUP treatment (average maximum dose = 0.7 mg/day), with the most relevant decline observed in the first three weeks. Moreover, Karp et al. showed an important improvement in executive functions and learning from pre- to post-treatment.

We then assessed five randomized, double-blind, placebo-controlled clinical trials (the studies of Emrich et al. [41], Dean et al. [49], Ehrich and colleagues [44], Yovell et al. [51], and Fava and colleagues [45]). Emrich et al. [41], through a pilot ABA study conducted in TRD patients treated with BUP, found a slight to strong reduction of depressive symptoms. Dean and colleagues [49], comparing BUP and methadone treatments on a group of heroin-dependent patients, reported improvements in depressive symptoms in all subjects, with no significant differences between the groups. Ehrich and colleagues [44] evaluated the efficacy of two different BUP/samidorphan combinations (8:1 dose-ratio = BUP/SAM 2 mg/0.25 mg for 3 days and BUP/SAM 4 mg/0.5 mg for four days; BUP:SAM 1:1 dose ratio = 4 mg/4 mg, and BUP/SAM 8 mg/8 mg over the same time periods), and observed the greatest antidepressant effects in the 1:1 ratio group, which resulted associated with the maximal blockade of opioid effects. Additionally, Fava and colleagues [45] evaluated the efficacy of this combination in MDD patients, in a four-week prospective study using BUP/SAM 2 mg/2 mg (the 2/2 dosage group) and BUP/SAM 8 mg/8 mg (the 8/8 dosage group), respectively. They found the greatest depression reduction in the 2/2 dosage group, while the improvement did not achieve statistical significance in the 8/8 dosage group.

Finally, Yovell et al. [51] observed a significant decrease in suicidal ideation in severely suicidal patients treated with an ultra-low-dose (mean final dosage = 0.44 mg/day) of sublingual BUP as an adjunctive treatment.

## 3. Discussion

### 3.1. Summary of Main Findings

All the considered (13) studies concluded that BUP—alone or in co-administration with opiate antagonists such as naloxone and samidorphan—may significantly reduce depression symptoms, NSSI, and suicidal ideation in both TRD patients and opiate-dependent patients. Importantly, Bodkin et al. [42] and Nyhuis et al. [46] reported some case reports regarding a complete remission with BUP. According to the studies of Striebel and Kalapatapu [47] and Ahmadi and colleagues [52], even considering that they are only clinical cases, a complete absence of suicidal ideation after BUP administration has been observed. Specifically, these effects were observed after one week of drug-treatment based on the study of Striebel and Kalapatapu [47], and after just few hours with a single BUP administration according to the Ahmadi and colleagues’ study [52]. Other studies including TRD patients, anyway, showed an improvement in depressive symptoms in different percentages: about 50% in Emrich et al. [41] and Fava et al. [45], and 66.7% in Karp and colleagues [43]. Regarding the studies on opioid-dependent patients, Kosten et al. [48] found a good response in 47% of the sample; in particular, 75% of responders showed the largest improvement during the first week of BUP treatment. Gerra and colleagues [50] observed similar results showing a significant reduction in the Symptom Checklist-90 (SCL-90) score of irritability, depression, tiredness, and psychosomatic symptoms after three months of BUP 4 mg + naltrexone 50 mg. Only in one study conducted on heroine dependent patients [49], BUP resulted as effective as methadone being associated with a significant clinical improvement on depressive symptoms, in methadone treated patients but fewer subjects on BUP remained depressed compared to those on methadone. However, as explained by the same authors, the doses of methadone and BUP may not have been equivalent in this study, making group comparisons difficult.

Overall, these results regarding the potential antidepressant activity of BUP, are in line with existing pharmacological studies where the agonism of MORs was correlated with increases in dopamine levels, enhancement of hedonic tone and sense of contentment, which together are responsible of the antidepressant activity [29]. Furthermore, post-mortem studies (conducted on depressed patients who committed suicide) revealed an increased µ-opioid receptor expression which may refer to an endogenous endorphin insufficiency [54], and can explain, at least partially, the efficacy of BUP on depression. Another explanation related to the antidepressant effects of BUP may be found in its monoamine inhibition activity [55]. In addition, BUP has also been shown to block the action of k agonists [56] and a functional k antagonism has been proposed on the basis of the results derived by animal models to function therapeutically as an antidepressant compound in humans [24] (this is the hypothesized mechanism underlying the efficacy of BUP/naltrexone combination therapy for opioid dependence [50]).

The results on the anti-suicidal potential of BUP are instead in line with both human and animal studies that linked suicidal behavior with both mental pain and endorphinergic control of the separation distress system [57]. Suicidal ideation and depression may be distinct, but related, phenomena (i.e., a reduction in depressive symptoms may accompany a reduction in suicidal ideation, as in ketamine infusion where improvements in suicidal ideation are related to, but not completely driven by, improvements in depression and anxiety [58]).

Regarding NSSI behavior, Norelli et al. [53] found an efficacy of BUP in reducing this behavior in five of the six investigated patients with long histories of severe repetitive NSSI behaviors refractory to conventional approaches. Importantly, in this study, different dosages of BUP and different treatment periods are used: case 1: BUP/naltrexone 0.5 mg/3 mg daily for nine months; case 2: BUP alone 4 mg/die for three months; case 3: BUP 2 mg/die for seven months; case 4: neither the dose nor the time was specified; case 5: BUP for 14 days—no indications about dosages; case 6: BUP 2–4 mg/die for seven months. Patients showed a significant reduction in NSSI behavior and, in two cases, the rapid improvement persisted up to one year even 12 months after discontinuation.

According to specific studies [22,45], BUP in co-administration with opiate antagonists, such as samidorphan, are able to significantly reduce depression symptoms, NSSI, and suicidal ideation.

The combination of BUP and potent µ-opioid antagonist with high sublingual bioavailability such as samidorphan (SAM) was initially investigated in opioid-experienced, non-depressed subjects. Later, a study assessed the preliminary efficacy of the combination BUP/SAM at the ratios identified in the first study as an adjunctive treatment in depressed individuals having an inadequate response to SSRIs or serotonin–norepinephrine reuptake inhibitors (SNRIs). Overall, the agonism of µ-opioid receptors was correlated with a consistent increase in dopamine levels, enhanced hedonic tone, and sense of contentment, while the safety and tolerability profile of BUP/SAM was favorable. Importantly, the use of both an opioid agonist/antagonist with opposing pharmacological action of similar magnitude, able to exert a balanced agonist–antagonist opioid modulation, seems to be linked with a normalization of the dysregulated/impaired opioidergic tone and may yield therapeutic benefits in major depression.

Taken together, according to the included studies, BUP has a rapid antidepressant and anti-suicidal action: in fact, this psychoactive compound seems to act in about a week after the first administration [41,42,43,44,46,47,48,52]. This rapid response resembles the time course of sleep deprivation and electro-convulsive therapy (ECT); however, the antidepressant effects of BUP might also suggest other mechanisms of action than that regarding current available antidepressants, for instance the activation of endorphin release in the central nervous system similarly to what occurred after electroconvulsive therapy (ECT) [59].

Even after ketamine treatment, which is an NMDA receptor antagonist binding to opioid µ and sigma receptors, rapid antidepressant effects together with a rapid reduction of suicidal behavior in depressed individuals were observed based on human studies. Similarly to ketamine, BUP may exert a rapid neuroplastic activity (ketamine, probably through the brain-derived neurotrophic factor (BDFN), can induce enhanced dendritic branching and synaptic receptor number and density) [7].

On the other hand, there are also studies [42,47,48,50,51] suggesting that this action is prolonged in time up to one to three months. According to the studies conducted by Emrich et al. [41] and Karp et al. [43], once the drug is discontinued after a short-term therapy (e.g., one week), the total score scales assessing depressive symptoms such as HAM-D and MADRS rapidly increased, suggesting that the improvement in depressive symptoms may require long-term BUP treatment to be sustained.

Only the study of Karp et al. [43] investigated the effect of BUP on specific cognitive functions (i.e., inhibition control, psychomotor speed, and memory for new information) in over 50 patients who were treated with a low-dose of BUP for two months. These cognitive domains did not worsen during exposure to the drug. It is well known that the effective treatment of depression may be also associated with a general improvement of cognitive symptoms (e.g., pseudo-dementia) [60]. However, high dose opioids are linked to a worsening of cognitive functions [61], although not in non-opioid-naïve patients [62]. In the study of Karp et al. [43], the lack of slowed (and potentially improved) psychomotor speed, inhibition, and memory suggests that low-dose BUP does not worsen, and may potentially improve, cognitive functions.

Based on the available evidence, there is no uniqueness associated with the better formulation to be used, neither regarding the dosage nor the expected treatment time. Generally, three types of preparations have been used: sublingual or intranasal BUP alone, BUP/SAM, BUP/NAL, or BUP/naltrexone. For the latter formulation, a simultaneous administration of an opioid agonist and antagonist, may be sufficient to normalize the dysregulated endogenous opioidergic tone in the context of depression [44]. Additionally, this type of association—whether in a balanced ratio (i.e., 1:1, 2:2 etc.) which is associated with maximal blockade of opioid effects—can prevent the potential additive properties of BUP [44].

Many studies [44,45,49,50,51] proposed the treatment with BUP in augmentation with standard antidepressants such as SSRIs or SNRIs (or with other drugs, such as antipsychotics, benzodiazepines, and mood stabilizers [51,52] with which the patient was usually being treated). The main used dosages ranged from very low- (0.1–0.2 mg) to high dosages (8 mg) while in just one case—a woman with severe opioid use disorder in comorbidity with TRD and severe suicidal behavior [47]—BUP was titrated up to 16 mg. Treatment time starts from four days and goes up to three months, and—as already highlighted above—only in the studies where BUP was administered for a long period, the results obtained were maintained. In particular, Kosten et al. found that depressive symptoms, in opioid-addicted patients on BUP treatment, are consistently reduced for a month with a slight plateau in the second week.

Not all the studies included in this review analyzed the occurrence of side effects related to BUP use but, in those who considered their possible emergence [42,43,44,45,46,50,51], specific side-effects were described in the majority of patients. In line with what was expected, the most common side effects of opiate drugs include: (1) nausea; (2) constipation; (3) sedation; (4) dizziness; (5) fatigue; (6) headache; (7) dry mouth; and (8) hyperhidrosis. These symptoms were of mild intensity, dose dependent, and transient (they seemed to last only for the first few days of treatment) and they may be avoided with a slower titration. Bodkin and colleagues [42] and Gerra et al. [50] even reported some cases of irritability, anxiety, and dysphoria, which led to drop-out from the study of implicated patients. Withdrawal symptoms were also analyzed in the studies of Karp et al. (0.2–1.6 mg BUP for eight weeks) [43], Fava et al. (BUP/SAM 2/2 or 8/8 mg) [45], Yovell and colleagues (0.1–0.2 mg/die BUP for four weeks) [51] but they were not reported after one week of drug discontinuation—or later.

### 3.2. Main Shortcomings/Limitations

The present systematic review needs to be interpreted in the light of the following limitations. First, most of the studies involve relatively disparate dosage regimens, questionable persistence of antidepressant effects and mixed methods for the analysis of the data that do not allow us to conduct a meta-analysis upon the main topic.

In addition, the short duration of the study designs of some studies [41,44,46,52] need to be mentioned. Unfortunately, a period of one week [41,44,46], or a few days more [52], may be not sufficient to observe the long-term efficacy or the emergence of some adverse effects related to this agent.

Another important shortcoming is represented by the modest sample size of almost all the samples (with the exclusion of some studies [45,50,51]) which does not allow to obtain a good statistical significance and, hence, does not allow the generalization of the main findings. Importantly, due to the small number of findings regarding the main topic in the investigated literature, we included in this systematic review even three case reports, the results of which are difficult to be generalized.

Another limitation in evaluating these studies was the heterogeneity of the clinical diagnoses related to the included subjects. Not all studies considered samples of patients with major depression or TRD, or even samples of patients with substance-naïve patient studies along with studies of opioid-dependent patients who might have secondary depression (e.g., substance-induced). The study of Yovell et al. [51] also included individuals not suffering from MDD, but from other psychiatric conditions, such as borderline personality disorder (BPD), in which suicidal ideation was present. In addition, the participants in the study conducted by Norelli et al. [53] showed a complex history of severe and recurrent traumas, high rate of hospitalization, and a suboptimal response to various combinations of medications (e.g., antipsychotic medications including clozapine, antidepressants, mood stabilizers including lithium, valproate, and carbamazepine, benzodiazepines, α-adrenergic and β-blockers, and the opiate antagonist naltrexone). Current diagnoses were not clearly reported within this study, which listed several lifetime diagnoses per patient starting from those given in the infancy (i.e., attachment disorder of childhood, oppositional defiant disorder, impulse control disorder, conduct disorder, pervasive developmental disorder, attention deficit hyperactivity disorder, MDD without psychotic features, depressive disorder not otherwise specified, chronic post-traumatic stress disorder (PTSD), BPD, borderline intellectual functioning, personality disorder not otherwise specified, schizoaffective disorder, substance abuse, anorexia and bulimia, dissociative disorder, and paraphilia). Therefore, this group of patients appeared to be extensively heterogeneous and there seems to be no unanimity in the interpretation of the symptoms for diagnostic purposes.

Overall, according to the myriad of clinical diagnoses, the wide-range of dosages and variable pharmacological formulations, an adequate comparison among studies is not feasible. Finally, it should also be noted that, except for the studies of Fava et al. [45], Yovell and colleagues [51], Ehrich et al. [44] and Dean et al. [49], the other nine studies lacked a control group.

## 4. Materials and Methods

### 4.1. Eligibility Criteria

In order to achieve a high standard of reporting, we adopted the “Preferred Reporting Items for Systematic Reviews and Meta-Analyses” (PRISMA) guidelines [63]. We included studies that explicitly mentioned the association between buprenorphine AND depression (OR TRD OR refractory depression), OR buprenorphine AND suicid* (including suicidal ideation OR suicidal thoughts OR suicide attempts), in clinical samples. We also included three studies that investigated depression in opiate dependent patients (secondarily exploring the efficacy of BUP on depressive symptoms). We excluded studies that explicitly investigated the use of BUP as an analgesic compound. When a title or abstract seemed to describe a study eligible for inclusion, the full-text article was obtained and carefully examined by a senior author (GS) to assess its relevance for the inclusion in our review. Specifically, our exclusion criteria were as follows: (1) studies published before 1980; (2) studies without abstracts or with abstracts that did not explicitly mention the association between depression/suicidal behavior and BUP; (3) studies that were not published in English; and (4) systematic reviews or meta-analytic studies regarding the main topic.

### 4.2. Information Sources

We conducted a systematic search of two major electronic databases comprising medical and social science studies (PubMed and Scopus were used, while we did not search Psychinfo because the search did not produce any consistent results concerning the main topic, and Science Direct was not used in order to reduce redundancy) for titles and abstracts (January 1980–June 2018) relevant for our research question. We additionally reviewed bibliographies from retrieved articles for additional papers that might be relevant to explore the main topic of this research.

### 4.3. Search Terms

The following search query was used in Pubmed: “buprenorphine” AND “depression” AND “suicid*” AND “treatment-resistant depression” AND “TRD” AND “refractory depression”. In addition, the search query that was used in Scopus was as follows: TITLE-ABS-KEY (buprenorphine) TITLE-ABS-KEY (suicid*) AND TITLE-ABS-KEY (depression) AND TITLE-ABS-KEY (treatment-resistant depression) AND TITLE-ABS-KEY (refractory depression) AND TITLE-ABS-KEY (TRD).

### 4.4. Selection of Studies

Articles were screened and selected in a two-step process to minimize bias. First, two independent researchers (G.A., G.C.) conducted the literature search. Any discrepancies between the two reviewers who, blind to each other, examined the studies for possible inclusion were solved by consultation with the senior reviewers (G.S., M.A., H.N.). In the second phase, full-text articles that met our inclusion criteria were retrieved and independently reviewed by other investigators (M.P., D.D.B.) who discussed the design and characteristics of the studies to test whether the considered studies could be included in the review. If doubts remained, the study was put on the list of those awaiting assessment, pending acquisition of more information, and was then carefully re-analyzed for possible inclusion. Any disagreements in this step were solved by discussion between reviewers. 

### 4.5. Data Collection Process

A data extraction document was developed. Overall, G.A. and G.C. independently extracted the following data elements from the 13 studies included of this review (see ‘study sample’ below): author(s) and publication year, presence/absence of a control group, psychiatric diagnosis, study design, sample size and characteristics, treatment type, psychometric instruments, inter-reliability test, limitations, main conclusions (for more details, see Table 1, Table 2, Table 3 and Table 4). Reviewers acquired the full-text of all the 13 included articles. The principal reviewers (G.S., M.A., H.N.) analyzed independently all the studies and the other investigators (M.P., D.D.B.) finally reviewed the included documents.

## 5. Conclusions

In summary, despite the limitations mentioned above, it is possible to tentatively conclude that BUP is an effective, well-tolerated, sufficiently safe (at low doses) compound in reducing depressive symptoms and serious suicidal ideation, even in patients with TRD who do not respond to conventional antidepressant medications or ECT. However, further studies, in particular concerning the long-term efficacy and safety of this medication, need to be carried out, even due to its potential for abuse.

Given the efficacy at very low doses, BUP administration may be started at 0.1–0.2 mg daily and—if possible—it should be recommended to slowly titrate the drug, in order to avoid its side effects until a personalized dosage may allow a sufficient clinical response. Future studies, involving a larger number of patients, are required to replicate the preliminary antidepressant and antisuicidal properties of BUP.

Finally, additional studies are especially needed to test the efficacy of BUP vs. BUP + SAM vs. BUP + NAL vs. placebo, to assess both BUP’s clinical effect as well as to evaluate the withdrawal symptoms and the low risk of addiction with BUP at very low doses. 

## Figures and Tables

**Table 1:** Summary of the most relevant case-report studies concerning BUP and depression/suicidal behavior included in the present review.

Reference	Control Group	Diagnosis	Study Design	Sample	Treatment	Psychometric Instruments	Inter-Reliability Test	Limitations	Main Conclusions	Statistical Analyses	Quality Assessment
Norelli et al., 2013 [53]	No	Treatment resistant NSSI	Case-report	6 adults	Personalized doses of buprenorphine.	No instruments were used for different amounts of time	No	Short duration of the clinical study; small number of the evaluated subjects; lack of control group; exiguity of the experimental group; lack of exclusion criteria.	Five patients had a significant reduction in total incidents, seclusion and restraint episodes, NSSI and improved mood states. One patient had not statistically significant changes.	A comparison was made between the mean monthly number of overall incidents, NSSI episodes, and S/R episodes without buprenorphine treatment, and the average number with buprenorphine treatment. A *t*-test comparison was used to determine the significance of the differences between these two data sets. A t-value was calculated for the overall combined data from all patients, and each individual patient in the study. A t-value was calculated by dividing the overall increase or decrease from the baseline over the standard deviation of post-treatment data divided by the square root of the number of post-treatment data points.	I = 0; II = 0 III = 2; IV = 0 V = 0; VI = 0 Total score = 2
Striebel and Kalapatapu, 2014 [47]	No	Chronic suicidal ideation; TRD; Chronic back pain; Opioid dependence	Case-report	1 adult	16/4 mg of buprenorphine/naloxone; 3 month.	No instruments were used	No	Small number of the evaluated subjects; lack of control group; lack of exclusion criteria; lack of standardized measures.	The patient showed a reduction and cessation of suicidal thoughts and depression and a decrease of pain.	-	I = 0; II = 0 III = 1; IV = 0 V = 0; VI = 0 Total score = 1
Ahmadi et al., 2017 [52]	No	Chronic suicidal ideation due to substance-induced depressive disorder	Case-report	25-year old man	8-mg single dose of sublingual buprenorphine; 10 days.	BSIS; BDI	No	Short duration of the clinical study; small number of the evaluated subjects; lack of control group; exiguity of the experimental group; lack of exclusion criteria.	The patient had a rapid reduction and cessation of suicidal thoughts and depression.	-	I = 0; II = 0 III = 1; IV = 2 V = 0; VI = 0 Total score = 3

**Table 2:** Summary of the most relevant prospective studies concerning BUP and depression/suicidal behavior included in the present review.

Reference	Control Group	Diagnosis	Study Design	Sample	Treatment	Psychometric Instruments	Inter-Reliability Test	Limitations	Main Conclusions	Statistical Analyses	Quality Assessment
Kosten et al., 1990 [48]	No	Opioid addiction	Observational prospective study	40 adults	Buprenorphine 3.2 mg, with a range from 2 to 8 mg; 1 month.	BDI; SDS	No	Short duration of the clinical study; lack of control group; exiguity of the experimental group; diagnostic heterogeneity of the study population. Depression was not a primary outcome; self-report measures; lack of control group; short duration of the clinical study.	Depressive patients had a significant reduction in depressive symptoms at the end of the first week; this reduction continued over the second week. Depressive symptoms steadily declined during the month.	ANOVA	I = 2; II = 0 III = 1; IV = 1 V = 0; VI = 0 Total score = 2
Gerra et al., 2006 [50]	Yes	Opioid dependence	Controlled observational prospective study	60 adults	30 patients: naltrexone alone; 30 patients: naltrexone (50 mg oral dose) plus buprenorphine (4 mg sublingual); 12 weeks.	SCL-90; VAS for craving scores	No	Lack of randomization; lack of placebo buprenorphine control; diagnostic heterogeneity of the study population; lack of psychiatric evaluation at baseline; depression not as the primary outcome; SCL-90 is not specific for depression; lack of exclusion criteria; for psychopathological evaluations only self-report instruments were used; short duration of the clinical study.	Patients in the naltrexone plus buprenorphine group showed a greater reduction in irritability, depression, tiredness, psychosomatic symptoms and craving scores than patients in the naltrexone group. Patients of both groups showed a significant decrease of irritability, depression, tiredness and psychosomatic symptoms scores.	ANOVA; Kaplan-Meier; chi-square test.	I = 2; II = 1 III = 1; IV = 1 V = 0; VI = 0 Total score = 5
Nyhuis et al., 2008 [46]	No	TRD	Observational prospective study	6 adults	Buprenorphine ranging from 0.8 to 2.0 mg; 7 days.	HAM-D; BDI	No	Short duration of the clinical study; lack of control group; exiguity of the experimental group; lack of exclusion criteria.	All six depressive patients improved over one week; five patients reached a complete remission	-	I = 1; II = 0 III = 1; IV = 2 V = 0; VI = 0 Total score = 4

**Table 3:** Summary of the most relevant open label non-controlled clinical trials concerning BUP and depression/suicidal behavior included in the present review.

Reference	Control Group	Diagnosis	Study Design	Sample	Treatment	Psychometric Instruments	Inter-Reliability Test	Limitations	Main Conclusions	Statistical Analyses	Quality Assessment
Bodkin et al., 1994 [42]	No	TRD	Open label clinical trial, case report	10 adults	Buprenorphine. Dosage was titrated according to tolerance and clinical benefit, with a maximum daily dosage of 1.8 mg; 4-6 weeks.	HAM-D; ADDS; POMS; GAS	No	Short duration of the clinical study; small number of the evaluated subjects; lack of control group, exiguity of the experimental group.	Patients showed a clinically striking improvement in both subjective and objective measures of depression. Four patients achieved a complete remission of symptoms, two were moderately improved, and one deteriorated.	Paired *t*-tests.	I = 1; II = 0 III = 1; IV = 2 V = 0; VI = 0 Total score = 4
Karp et al., 2014 [43]	No	TRD	Open label clinical trial	15 adults aged 50 and older	Buprenorphine (from 0.2 mg to 1.6 mg/day). The average daily dose was 0.40 mg/day; 8 weeks.	MADRS; SCID; BSI-anxiety; the Positive and Negative Affect Scales; SSI; Choice Reaction Time Task Congruous vs. Incongruous Conditions Reaction Time Task; HVLT-R; UKU; FIBSER; SF-26; PSQI; COWS; MMSE	No	Lack of control group; exiguity of the experimental group; lack of randomization; short duration of the clinical study.	Patients exhibited a sharp decline in depression severity during the first 3 weeks, in particular in pessimism and sadness scores. Executive function and learning improved from pre- to post-treatment.	Descriptive analysis; Exact Wilcoxon tests.	I = 1; II = 0 III = 1; IV = 2 V = 0; VI = 0 Total score = 4

**Table 4:** Summary of the most relevant randomized, double-blind, placebo-controlled clinical trials concerning BUP and depression/suicidal behavior included in the present review.

Reference	Control Group	Diagnosis	Study Design	Sample	Treatment	Psychometric Instruments	Inter-Reliability Test	Limitations	Main Conclusions	Statistical Analyses	Quality Assessment
Emrich et al., 1982 [41]	Yes	TRD	Double-blind, placebo-controlled clinical trial	10 adults	Buprenorphine 2 mg per day; 4–8 days.	HAM-D; IMPS; VBS	No	Short duration of the clinical study; lack of control group; exiguity of the experimental group.	Overall, four patients showed more than 50% reduction in depression, two patients showed a moderate response, and four, a slight reduction.	Wilcoxon-test.	I = 1; II = 1 III = 1; IV = 2 V = 0; VI = 0 Total score = 5
Dean et al., 2004 [49]	Yes	Heroin-dependence	Randomized, double-blind, placebo controlled clinical trial	147 adults	68 patients: buprenorphine sublingual tablets and placebo methadone syrup; 79 patients: methadone syrup and placebo buprenorphine tablet. Dosing was initiated at 30 mg methadone or 4 mg buprenorphine; doses were individually titrated to optimize response; 3 months.	BDI	No	Diagnostic heterogeneity of the study population; self-scored questionnaires; lack of psychiatric evaluation at baseline; depression not as the primary outcome; lack of exclusion criteria.	Depressive symptoms improved in all subjects, with no difference between methadone and buprenorphine groups.	*t*-tests; chi-square tests. Treatment effects data were analyzed using a two-way fixed effects analysis of variance. Outcome predictors were examined using regression analyses.	I = 2; II = 2 III = 1; IV = 1 V = 0; VI = 0 Total score = 6
Ehrich et al., 2015 [44]	Yes	MDD and inadequate response to standard antidepressant therapy (TRD)	Randomized, double-blind, placebo controlled clinical trial	32 adults	14 patients: buprenorphine: samidorphan 8:1 dose-ratio; 14 patients: buprenorphine: samidorphan 1:1 dose-ratio; 4 patients: placebo; 1 week.	HAM-D; MADRS; VAS	No	Short duration of the clinical study; small number of the evaluated subjects; VAS not validated in this population; for psychopathological evaluations only self-report instruments were used.	Patients in the 1:1 ratio group in seven days exhibited statistically significant improvement in depressive symptoms; antidepressant effects were greatest in this group.	Descriptive statistics about safety, *t*-test; Bonferroni adjustment; Exact Wilcoxon tests.	I = 1; II = 1 III = 1; IV = 1 V = 0; VI = 0 Total score = 4
Yovell et al., 2016 [51]	Yes	Clinically significant suicidal ideation	Randomized, double blind, placebo-controlled clinical trial	88 adults	57 patients: buprenorphine (0.1 or 0.2 mg/day. Once a week, the daily dose could be raised of 0.1–0.2 mg increments); 31 patients: placebo; 4 weeks.	BSSI; BDI; SPS	No	Self-scored questionnaires; diagnostic heterogeneity of the study population.	Patients in the buprenorphine group had a greater reduction in suicidal ideation, suicide probability and depression scores than patients in placebo group.	Two-sided *t* tests for continuous variables and Pearson’s chi-square test or Fisher’s exact test, as appropriate, for categorical variables. Correlations were calculated using Pearson’sr. Fisher’s r-to-z transformation was used for testing the difference between correlations.	I = 2; II = 1 III = 1; IV = 1; V = 0; VI = 0 Total score = 5
Fava et al., 2016 [45]	Yes	MDD adults who had an inadequate response to one or two courses of antidepressant treatment (TRD)	Randomized, double-blind, placebo-controlled trial	142 adults	Buprenorphine/samidorphan 2 mg/2 mg; buprenorphine/samidorphan 8 mg/8 mg; placebo; 4 weeks.	HAM-D; MADRS; CGI-S	Yes	Short duration of the clinical study.	Patients in the 2:2 ratio group, compared with patients in the placebo group, showed significantly greater improvements. There was also evidence of improvement in the 8:8 ratio group although it did not achieve statistical significance.	The primary efficacy endpoint, was evaluated using the weighted combination of statistics from the stage-specific mixed models for repeated measures (MMRM); Kenward-Roger approximation was used to adjust the denominator degrees of freedom. Combined inference was conducted using the weighted linear combination of stage-wise test statistics.	I = 2; II = 2 III = 1; IV = 2 V = 2; VI = 2 Total score = 11

**Abbreviations:** MDD = Major Depressive Disorder; TRD = Treatment-Resistant Depression; BD = Bipolar Disorder; MADRS= Montgomery–Åsberg Depression Rating Scale; SCID-I = Structured Clinical Interview for DSM; BDI = Beck Depression Inventory; BSSI = Beck Scale for Suicidal Ideation; HAM-D = Hamilton Rating Scale for Depression; SCL-90 = Symptom Checklist-90; VAS = Visual Analogue Scale; BSI-Anxiety = Brief Symptom Inventory—Anxiety Subscale; UKU = Udvalg for Kliniske Undersogelser Side Effects Rating Scale; SF-36 = Short Form 36; FISBER = Burden of Side Effects Rating; PSQI = Pittsburgh Sleep Quality Index; COWS = Clinical Opiate Withdrawal Scale; HVLT-R = Hopkins Verbal Learning Test-Revised; MMSE = Mini Mental State Exam; SPS = Suicide Probability Scale; CESD = Center for Epidemiologic Studies Depression scale; BPI = Brief Pain Inventory; PHQ-2 = Patient Health Questionnaire; BPI = Brief Pain Inventory; SF-36v2 = Short Form version 2; MOS-SS = Medical Outcomes Study Sleep Scale; ODI = Oswestry Disability Index; ADDS = the Atypical Depression Diagnostic Scale; POMS = the Profile of Mood States; GAS = Global Assessment Scale; VBS = Verlaufs-Beurteilungs-Skala; CGI-S = Clinical Global Impressions severity scale; SDS = Short Depression Scale; IMPS = Inpatient Multidimensional Psychiatric Scale; BSIS = Beck Suicide Intent Scale; NSI = Non-Suicidal Self-Injury. **Quality assessment parameters**: (1) representativeness of the sample from the general population (0–2 points); (2) presence and representativeness of a control group (0–2 points); (3) presence of follow-up (0–2 points); (4) evidence based measures to evaluate suicidal ideation or suicide attempts (e.g., Suicidal Probability Scale—SPS, Suicidal Ideation Questionnaire—SIQ, Beck Hopelessness Scale—BHS, or other psychometric evaluations) or major depression (e.g., Montgomery Åsberg Depression Rating Scale—MADRS, Hamilton Depression Rating Scale—HDRS, or other psychometric evaluations) or concerning TRD, the use of rating scales for staging (e.g., Thase & Rush criteria, Souery criteria, etc.) (0–2 points); (5) presence of raters who identified independently the presence of suicidal ideation or suicide attempts or depression; (6) statistical evaluation of inter-rater reliability (0–2 points); (7) quality of the statistical analysis. Quality scores ranged from 0 to 14. Studies were assessed regarding quality as follows: (1) good quality (9–14 points) if most or all the criteria were fulfilled, or the study conclusions were deemed very robust; (2) moderate quality (4–8 points) whether only some criteria were fulfilled, or the study conclusions were deemed robust; and (3) low quality (0–3 points) whether few criteria were fulfilled or the conclusions of the study were not deemed robust. Caution was exercised in interpreting the findings related to low-quality studies.
